# Real‐time cardiac cine MRI: A comparison of a diffusion probabilistic model with alternative state‐of‐the‐art image reconstruction techniques for undersampled spiral acquisitions

**DOI:** 10.1002/mrm.30572

**Published:** 2025-06-16

**Authors:** Oliver Schad, Julius Frederik Heidenreich, Nils Petri, Jonas Kleineisel, Simon Sauer, Thorsten Alexander Bley, Peter Nordbeck, Bernhard Petritsch, Tobias Wech

**Affiliations:** ^1^ Department of Diagnostic and Interventional Radiology University Hospital Würzburg Würzburg Germany; ^2^ Department of Internal Medicine I University Hospital Würzburg Würzburg Germany; ^3^ Comprehensive Heart Failure Center Würzburg Würzburg Germany

**Keywords:** cardiac imaging, diffusion models, heart, machine learning, magnetic resonance imaging, variational network

## Abstract

**Purpose:**

Electrocardiogram (ECG)‐gated cine imaging in breath‐hold enables high‐quality diagnostics in most patients but can be compromised by arrhythmia and inability to hold breath. Real‐time cardiac MRI offers faster and robust exams without these limitations. To achieve sufficient acceleration, advanced reconstruction methods, which transfer data into high‐quality images, are required.

**Methods:**

In this study, undersampled spiral balanced SSFP (bSSFP) real‐time data in free‐breathing were acquired at 1.5T in 16 healthy volunteers and five arrhythmic patients, with ECG‐gated Cartesian cine in breath‐hold serving as clinical reference. Image reconstructions were performed using a tailored and specifically trained score‐based diffusion model, compared to a variational network and different compressed sensing approaches. The techniques were assessed using an expert reader study, scalar metric calculations, difference images against a segmented reference, and Bland–Altman analysis of cardiac functional parameters.

**Results:**

In participants with irregular RR‐cycles, spiral real‐time acquisitions showed superior image quality compared to the clinical reference. Quantitative and qualitative metrics indicate enhanced image quality of the diffusion model in comparison to the alternative reconstruction methods, although improvements over the variational network were minor. Slightly higher ejection fractions for the real‐time diffusion reconstructions were exhibited relative to the clinical references with a bias of 1.1 ± 5.7% for healthy subjects.

**Conclusion:**

The proposed real‐time technique enables free‐breathing acquisitions of spatio‐temporal images with high quality, covering the entire heart in less than 1 min. Evaluation of ejection fraction using the ECG‐gated reference can be vulnerable to arrhythmia and averaging effects, highlighting the need for real‐time approaches. Prolonged inference times and stochastic variability of the diffusion reconstruction represent obstacles to overcome for clinical translation.

## 1 INTRODUCTION

Real‐time acquisition instead of segmented k‐space sampling is advantageous for cardiovascular MRI in many respects.^1^ First and foremost, motion artifacts, especially in patients with arrhythmia or those with problems to hold breath, can be greatly reduced by avoiding the averaging of information across several heartbeats.^2^ But even in compliant patients, comfort can be considerably improved eliminating breath‐holds, electrocardiogram (ECG) gating, and reducing scan times. The increased efficiency can reduce costs and indirectly open access to cardiac MRI for a broader patient population.^3^ Moreover, the accuracy of investigating dynamic cardiac motion could be enhanced if data were not pooled from multiple RR‐cycles, which are inherently variable and may be suboptimally gated.^4^

To provide comparable spatial and temporal resolution as typically realized by segmented cine scans, real‐time cardiac MRI (cMRI) requires fast data acquisitions. High performance gradient systems and pulse sequences with short TRs, such as balanced SSFP (bSSFP), as well as non‐Cartesian k‐space trajectories are advantageous for the needed efficiency.^5^ The use of phased‐array coils enables the application of parallel imaging and, thus, to sample below the Nyquist‐rate. Additional regularization by sparsity models or more recently by means of data‐driven priors have further promoted decent image quality despite significant undersampling rates.^5^ Especially in iterative reconstructions, precise knowledge of the k‐space trajectory is crucial. Under realistic scan conditions, confounding factors like eddy currents can cause temporal deviations from the intended gradient waveform. For Cartesian imaging, this typically results in a shift in k‐space, leading to a linear phase within image space. In radial or spiral sampling, however, shifted projections or arms can interfere with each other in the oversampled center of k‐space, potentially causing more significant artifacts. Repeatedly enforcing consistency with these corrupted data can exacerbate the errors. To this end, correcting trajectories, for example with a gradient system transfer function, has proven to raise image quality.^6^, ^7^

Nevertheless, clinical protocols to assess cardiac function etc. are still often dominated by segmented acquisitions. This might be due to the fact that image quality is ‐ on average ‐ often superior compared to state‐of‐the‐art (SOTA) real‐time alternatives.

In this paper, we therefore compare established parallel imaging‐ and compressed sensing (CS)‐based models, a variational network (VN) as well as more recently introduced score‐based generative diffusion models^8^ for the reconstruction of functional cardiac MRI scans based on efficient spiral k‐space sampling.

First adapted for undersampled MR reconstruction by Hammernik et al.,^9^ VNs utilize an unrolled network architecture, which alternates between data consistency, (deep) neural network‐based denoising, and artifact removal blocks. A prior in the data reconstruction is induced by pairing artifact‐afflicted data with a corresponding ground‐truth. First implementations employed basic convolutional neural network (CNN) architectures,^10^ followed by more advanced neural network architectures such as U‐Nets,^11^ recurrent neural networks,^12^ or transformer back‐bones.^13^ Further developments demonstrated VNs fully trained end‐to‐end^11^ along with the integration of gridding operations in the unrolled blocks for non‐Cartesian reconstructions, as presented by Kleineisel et al.^14^ for undersampled spiral acquisitions.

Alternatively, information regarding the underlying data distribution of artifact‐free high‐quality images can serve as a prior in the regularization of MR reconstruction algorithms independent of the imaging operator.^15^, ^16^ Techniques from generative modeling such as variational auto‐encoders (VAE), generative adversarial networks (GAN), and more recently diffusion probabilistic modeling have been explored. The capability of modeling complex structures using VAEs is often limited by the network complexity, leading to low‐fidelity samples. GANs can capture complex structures more effectively, but are prone to training instabilities. Nevertheless, also GANs are constantly improved, for example, with newer implementations achieving promising results using transformer backbone architectures.^17^, ^18^

Emerging diffusion probabilistic models allow learning of the data distribution prior in a straight forward, adversarial‐free manner due to the underlying fundamental mathematical basis. Being able to achieve stable and high‐quality image generation, they have also attracted attention for medical imaging, with first applications for accelerated MRI reconstruction showing promising results.^16^, ^19^, ^20^, ^21^

While previous works mainly exploited retrospectively undersampled data from openly available databases, here we train a diffusion model from scratch on spiral cardiac MR data. Consequently, we have the advantage that the trained network can approximate a target distribution in good accordance with our actual acquisition procedure.

We first describe a diffusion probabilistic approach^8^, ^20^, ^22^ to learn the distribution of the subspace of cine MRI scans with high fidelity and subsequently propose a method to exploit this information to regularize a physics driven reconstruction of real‐time cardiac acquisitions. In a study involving healthy volunteers and patients with arrhythmic cardiac diseases, the method is then compared against alternative techniques like VNs,^9^, ^11^, ^14^ a low rank plus sparse algorithm,^23^ as well as total variation‐ and wavelet‐based compressed sensing.^24^

## 2 METHODS

MRI reconstruction can be understood as a linear inverse problem y=Ax, where the image $x\in {\mathbb{C}}^{n\times m}$ has to be retrieved from the complex multi‐coil data $y\in {\mathbb{C}}^{{\#}_{\text{coils}}\times n\times m}$. Typically, the “MRI Operator” A can be disentangled into A=MFS, consisting of coil sensitivities $S\in {\mathbb{C}}^{{\#}_{\text{coils}}\times n\times m}$, the Fourier transform F and a sampling mask M. For acquisitions with multiple receiver coils (i.e. ${\#}_{\text{coils}}>1$), parallel imaging can be exploited to reconstruct images from undersampled acquisitions M, where the acceleration factor R leads to a violation of the Nyquist criterion in k‐space. However, as measurements with multiple coils are correlated, R can typically not nearly reach ${\#}_{\text{coils}}$, without significantly increasing noise or residual artifacts. In the case of non‐Cartesian sampling, F needs to incorporate some kind of gridding procedure to transfer data onto a Cartesian grid. We will use the term “naïve” to refer to the undersampled, gridded, and coil‐combined reconstructions without further regularizations. Coil‐combinations were performed using a coil‐sensitivity weighted sum $x_{\text{comb}}=\sum_{i=1}^{{\#}_{\text{coils}}}S_{i}^{*}x_{i}$, where $S_{i}^{*}$ are complex‐conjugate coil‐sensitivity maps and $x_{i}$ are coil‐images.^25^

To maximize acceleration potential, numerous reconstruction techniques have been proposed beyond parallel imaging, leveraging prior knowledge of the underlying data distribution and regularizing the inverse equation accordingly. This can be described by the following optimization problem

(1) $$x^{*}=\underset{x}{\arg\min}{\left\|A\;x–y\right\|}_{2}^{2}+\lambda \psi \left(x\right),$$

where ψ(x) represents a λ‐weighted regularization term.

In the following, we first establish our proposed acquisition scheme for accelerated (real‐time) cMRI based on spiral bSSFP. Subsequently, a reconstruction technique based on diffusion probabilistic models and the corresponding training is described. Ultimately, alternative modern reconstruction techniques are briefly outlined.

### 2.1 Data acquisition based on spiral waveforms

**FIGURE 1:**
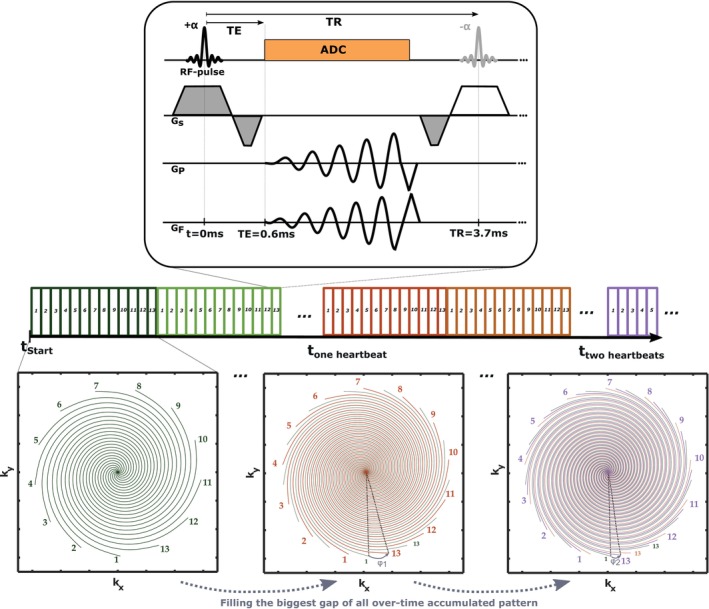
Overview of the spiral bSSFP‐sequence. Real‐time frames contain data from 13 equidistant spiral arms, which were repeatedly acquired for a duration slightly exceeding one RR‐cycle in breath‐hold (as checked by the ECG of the scanner). After one heartbeat, this pattern was rotated, filling the largest gap in between all previously captured patterns. Sampling for approximately nine heartbeats allowed for segmented reconstructions of reference cardiac phases in addition to real‐time acquisitions. For a shortened scan duration in free‐breathing acquisitions, the pattern was rotated already after a fraction of an RR‐cycle.

To provide fast and efficient data sampling for real‐time cardiac MRI, we implemented a spiral bSSFP‐sequence, schematically depicted by the pulse sequence diagram in Figure 1. Spiral trajectories were designed using the variable density spiral algorithm by B. Hargreaves,^26^ further optimized to account for peripheral nerve stimulations.^27^ Triangular rewinders were appended to the spiral read out to null the zeroth gradient moment. Trajectories were retrospectively corrected using the scanners gradient system transfer function as determined in a different study.^7^ Banding artifacts were avoided in the shimmed region of interest by using RF phase alternation of 180° (±α).

We acquired data in breath‐hold and free‐breathing. The former were acquired in healthy volunteers and used to obtain paired segmented (reference) and undersampled real‐time data for supervised trainings, and to evaluate the developed methods based on scalar image metrics. Additionally, segmented spiral data acquired in breath‐hold were used to train the unconditioned diffusion model.

Free‐breathing scans (undersampled, real‐time) represent the targeted application and were acquired both in healthy volunteers and patients, to assess image quality with respect to alternative methods in an expert reader study, and to test the accuracy in quantifying cardiac functional parameters.

#### 2.1.1 Acquisitions in breath‐hold – Providing segmented and real‐time data by a single scan

The ultimate aim of our method is to reconstruct undersampled real‐time frames, which are sampled one after the other and in free breathing. One such real‐time frame consisted of 13 successively acquired, equidistant spiral arms (FOV ˜ 9 cm toward the k‐space center, linearly decreasing to FOV ˜ 3 cm in the periphery) with a temporal resolution of ˜48 ms per frame (see Figure 1 and acquisition parameters in Table 1).

**TABLE 1:** Overview of the acquisition parameters of the breath‐held Cartesian cine reference and the accelerated free‐breathing spiral real‐time technique.

Sequence parameters	Segmented Cartesian cine bSSFP	Undersampled spiral real‐time bSSFP (13 spiral arms)
Slice thickness [mm]	8	8
Flip angle [°]	70	70
Spatial resolution [mm]	1.25 × 1.25	1.29 × 1.29
FOV [mm]	320 × 260	592 × 592
Image matrix [px]	256 × 208	512 × 512
TR [ms]	3.94	3.70
TE [ms]	1.97	0.61
Temporal resolution [ms]	44 ± 4	48
Pixel bandwidth [Hz/pixel]	528	407
GRAPPA	2	‐

*Note*: The theoretical spatial resolution of the undersampled spiral technique is given by the k‐space location with the largest distance to the k‐space center during acquisition. Since transferring off‐grid data to a 512 × 512px Cartesian grid using GROG introduced slight zero filling, reconstructions displayed pixel sizes of 1.16 × 1.16 mm with corresponding, reconstructed FOV.

To obtain a dataset which allows to build both images of cardiac phases in a segmented fashion (binning data over several RR‐cycles as for classical cine) and in real‐time (undersampled), acquisitions were first performed in healthy volunteers and under breath‐held conditions.

To enable this, the trajectory as shown in Figure 1 was subjected to a tailored rotation scheme for consecutive frames (see Video S1 for an extended illustration): The pattern of 13 equidistant arms (angle increment between individual spiral arms ˜28°, temporal footprint: 48 ms) was repeated exactly the same for a period of time slightly longer than one RR interval $t_{\mathrm{RR}}$. The latter was estimated directly before the acquisition using the scanner's “live” ECG. The number of repeatedly acquired patterns $N_{\text{cine}}$ was consequently chosen to fulfill $48\;\mathrm{ms}*N_{\text{cine}}≳t_{\mathrm{RR}}$.

After each such a block of duration $48\;\mathrm{ms}*N_{\text{cine}}$, that is repeating the pattern of 13 equidistant spiral arms $N_{\text{cine}}$ times, a “new” pattern was built by rotating the initial pattern as a whole by an angle φ. This pattern was then again used without rotation for another $N_{\text{cine}}$ repetitions, until another rotation was performed, and so on. φ was chosen to fill that the largest gap in the angular dimension of all acquisitions accumulated over time, that is $\varphi_{1}=\frac{2\pi }{13}*\frac{1}{2}$, $\varphi_{2}=\frac{2\pi }{13}*\frac{1}{4}$, $\varphi_{3}=\frac{2\pi }{13}*\frac{3}{4}$, $\varphi_{4}=\frac{2\pi }{13}*\frac{1}{8}$, $\varphi_{5}=\frac{2\pi }{13}*\frac{5}{8}$ etc.

This was proceeded for approximately nine heartbeats (total duration = $9*N_{\text{cine}}*48\;\mathrm{ms}$), balancing k‐space coverage with breath‐hold duration, and allowing for one transient‐phase heartbeat without rotation. Total scan times ranged from 9 to 15 s for a single slice. Repetitive sampling of the k‐space center (*k* = 0) enabled extraction of a self‐gating “ECG” signal (i.e., the “DC‐signal”).

With this sampling approach, data for individual cardiac phases can be mixed from multiple RR‐intervals (in units of 13 arms), that is “segmented cine imaging”, and, additionally, the same scan can still be regarded and reconstructed as real‐time, by building frames for each of the consecutive patterns of 13 spiral arms. By binning the differently rotated, undersampled real‐time frames from matching timestamps in the cardiac cycle using the self‐gating signal, $N_{\text{cine}}$ segmented cardiac phases were derived. These consist of 104 spiral arms with equidistant angular distribution and were used as spiral “aliasing free” references.

#### 2.1.2 Real‐time cardiac imaging in free breathing

Having trained and set up the different reconstruction approaches as detailed below, we assessed our approach in free‐breathing condition in both healthy volunteers and patients. Here, we acquired blocks of $N_{\text{cine}}=10$ repeated patterns without rotation, resulting in a total scan time for one slice of ˜5 s. Each of the nine blocks now does not exceed the length of an RR‐interval anymore. We have only refrained from rotating the pattern after every frame (i.e. $N_{\text{cine}}=1$) to avoid high‐frequent flickering of residual aliasing artifacts in the dynamic view. Throughout the entire acquisition, however, we are still able to build a temporal average with 104 equidistant arms.

All spiral acquisitions were initially transferred onto a Cartesian grid of size 512 × 512 px using GRAPPA Operator Gridding (GROG).^28^, ^29^ To this end, GRAPPA Kernels were calculated from a temporal average reconstruction using convolution gridding. In order to perform coil(de)combinations, coil sensitivity maps were computed from the temporally averaged k‐spaces using ESPIRIT^30^ as a part of BART.^24^

### 2.2 Acquisitions in healthy volunteers and patients suffering from atrial fibrillation

This prospective study was approved by the local ethics committee under license ID 173/22_skpm, and written informed consent was obtained from each participant. Inclusion criteria were age > 18 years, and for patients a clinically diagnosed intermittent atrial fibrillation. Exclusion criteria were common contraindications to MRI examinations.

A total of 16 healthy participants and five patients with atrial fibrillation diseases were examined on a 1.5T clinical whole‐body MR system (Magnetom Avanto^fit^, Siemens Healthcare, Erlangen, Germany), which provided a maximum gradient amplitude of 40 mT/m and a maximum slew rate of 170 mT/m/ms.

The data from eight healthy volunteers were exclusively used for training purposes (see descriptions below). The remaining eight volunteers and five patients were used to evaluate the imaging approaches (test data).

In each participant, ECG‐gated, breath‐held clinical Cartesian bSSFP acquisitions were performed as a reference, seamlessly covering the entire left ventricle from base to apex in a series of sequentially measured left‐ventricular short‐axis (SAX) slice orientations (9–13 slices).

For all healthy volunteers (both entire training data set and healthy volunteer split of the test data), breath‐held spiral acquisitions as depicted in Section 2.1, were scanned at identical slice positions. Training data were used to fit the neural networks used in our data‐driven reconstruction techniques; the test data served for deriving scalar image metrics during evaluation.

Ultimately, real‐time acquisitions in free breathing were obtained from all participants of the test data (eight healthy volunteers and five patients), again for the same slices.

An overview of the measurement parameters for both methods can be found in Table 1.

### 2.3 Score‐based diffusion models

#### 2.3.1 Vanilla score‐based diffusion models

We follow the description of the generative diffusion process using a score‐based approach^8^ to model the underlying data distribution of cMRI data. For a more detailed description of diffusion models and their usage as a prior for solving inverse problems, see.^8^, ^20^, ^22^, ^31^, ^32^

Consider a diffusion process for an image x(t), where t∈[0,T] presents a continuous time variable. x(0) corresponds to a sample of the probability distribution $p_{0}\left(x\right)$ of a given dataset and x(T) is the sample fully perturbed to Gaussian noise. Such a process demonstrates a solution to the stochastic differential equation (SDE) of the form: dx=f(x,t)dt+g(t)dw, where w corresponds to Brownian motion.

Reversing the given SDE by starting from Gaussian noise to obtain samples from the data distribution $p_{0}\left(x\right)$ also results in a diffusion process, with the reverse‐time SDE (rSDE) following

(2) $$\mathrm{dx}=\left[f\left(x,t\right)\;\mathrm{dt}-g{\left(t\right)}^{2}\;\nabla_{x}\log\left(p_{t}\left(x\right)\right)\right]\;\mathrm{dt}+g\left(t\right)\;{\mathrm{dw}}^{\prime }.$$

Choosing f(x,t)=0 and $g\left(t\right)=\sqrt{\frac{d\left(\sigma {\left(t\right)}^{2}\right)}{\mathrm{dt}}}$ results in so called variance exploding (VE) SDE, where σ(t) is a positive, monotonically increasing function, typically chosen to be a geometric sequence, such as $\sigma \left(t\right)=\sigma_{\min}{\left(\frac{\sigma_{\max}}{\sigma_{\min}}\right)}^{t}$ for t∈[0,1].

If the score $\nabla_{x}\log\left(p_{t}\left(x\right)\right)$ is defined for all timesteps t, one can generate samples from the distribution $p_{0}\left(x\right)$ by solving the rSDE.

Commonly, since the true score is not available, it is estimated by training a time‐dependent neural network $s_{\theta }\left(x\left(t\right),t\right)$ using denoising score matching^8^, ^20^, ^22^, ^33^, ^34^, ^35^, ^36^ and optimizing the following l2‐loss function

(3) $$\underset{\Theta }{\arg\min}E_{t,x\left(0\right),x\left(t\right)|x\left(0\right)}\left[\lambda \left(t\right){\left\|s_{\Theta }\left(x\left(t\right),t\right)-\nabla_{x_{t}}\log\left(p_{t}\left(x\left(t\right)|x\left(0\right)\right)\right)\right\|}_{2}^{2}\right].$$

Here λ(t) corresponds to a positive time‐dependent weighting function, that adds additional weighting for different time points to stabilize training.^37^ The perturbation in Eq. [3] of x(t) for a specific step t can be expressed by the unperturbed input $x_{0}$ and a single normal distributed kernel $p\left(x\left(t\right)|x\left(0\right)\right)=N\left(x\left(t\right)|x\left(0\right),\sigma {\left(t\right)}^{2}I\right)=\frac{1}{\sqrt{{\left(2\pi \right)}^{n}\sigma {\left(t\right)}^{2n}}}\exp\left[-\frac{{\left\|x\left(t\right)-x\left(0\right)\right\|}_{2}^{2}}{2\sigma {\left(t\right)}^{2}}\right]$, with dimension n. Calculating the gradient $-\nabla_{x_{t}}\log\left(p\left(x\left(t\right)|x\left(0\right)\right)\right)=\left(x\left(t\right)-x\left(0\right)\right)/\sigma {\left(t\right)}^{2}$ and using x(t)=x(0)+σ(t)z, the training objective simplifies to ${\arg\min}_{\Theta }E_{t,x\left(0\right),x\left(t\right)|x\left(0\right)}\left[\lambda_{i}{\left\|s_{\Theta }\left(x\left(t\right),t\right)+z/\sigma \left(t\right))\right\|}_{2}^{2}\right]$, where z˜N(0,I) is standard Gaussian noise. Similar to,^37^ choosing the weighting function $\lambda \left(t\right)=\sigma {\left(t\right)}^{2}$, the final training task simplifies to ${\arg\min}_{\Theta }E_{t,x\left(0\right),x\left(t\right)|x\left(0\right)}\left[{\left\|\sigma \left(t\right)s_{\Theta }\left(x\left(t\right),t\right)+z\right\|}_{2}^{2}\right]$.

After training the network as an approximation for the score, $\nabla_{x}\;\log\left(p_{t}\left(x\right)\right)\approx s_{\Theta }\left(x\left(t\right),t\right)$ is reinserted into Eq. [2], and the rSDE can be solved, for example by using numerical methods such as reverse diffusion and Euler‐Mayurama sampling, Langevin dynamics or hybrid algorithms such as predictor–corrector methods.^38^, ^39^, ^40^ To sample from the prior distribution, t is in practice typically uniformly discretized into *N* steps $t_{i}=\left(i-1\right)\Delta N$ with $\Delta N=\frac{1}{N-1}$ and i ∈[1,N], which leads to the continuous description in the case for N→∞. Thereby, new samples can be drawn from the learned data distribution. The discretized sampling process of the “reverse diffusion sampler” to solve Eq. [2], which we use in this work, is given by $x_{i-1}=x_{i}-f_{i}\left(x_{i}\right)+g_{i}g_{i}^{T}\;s_{\Theta }\left(x_{i},i\right)+g_{i}z$, with *z* being standard Gaussian noise as described by Song et al.^8^

#### 2.3.2 Conditioned sampling

So far, the description of the diffusion models solely applies for a purely generative task. However, solving an inverse problem translates to computing the posterior probability p(x(t)|y) for an image *x*(*t*) given the data *y*, which can be rewritten to p(x(t)|y)˜p(x(t))p(y|x(t)) using Bayes rule. According to Song et al.,^8^ computing the score corresponds to $\nabla_{x}\log\left(p_{t}\left(x\left(t\right)|y\right)\right)\approx \nabla_{x}\log\left(p_{t}\left(x\left(t\right)\right)\right)+\nabla_{x}\log\left(p_{t}\left(y\left(t\right)|x\left(t\right)\right)\right)=s_{\Theta }\left(x\left(t\right),t\right)+\nabla_{x}\log\left(p_{t}\left(y\left(t\right)|x\left(t\right)\right)\right).$ The first term resembles the unconditioned score function, estimated by the trained network. The latter term is typically not tractable, as only the dependence p(y|x(0)) is known.

Approximating $\nabla_{x}\log\left(p_{t}\left(y\left(t\right)|x\left(t\right)\right)\right)$ is an active research topic and a variety of sampling strategies for image restoration tasks have been proposed.^22^, ^31^, ^41^, ^42^, ^43^, ^44^ In this work, we use diffusion posterior sampling^31^ (DPS), which has proven a popular method for performing conditioned sampling. The key idea of DPS is to approximate the gradient of the log likelihood via $\nabla_{x_{t}}\log\left(p\left(y|x\left(t\right)\right)\right)\approx \nabla_{x_{t}}\log\left(p\left(y|{\hat{x}}_{0}\left(x\left(t\right)\right)\right)\right)$. Commonly described as the posterior mean, ${\hat{x}}_{0}$ corresponds to a denoised estimate of clean x(0) given noisy x(t) at intermediate reverse timesteps.

Tweedie's formula^45^ provides a way to estimate a denoised estimate from the noisy observation through ${\hat{x}}_{0}\left(x\left(t\right)\right)=E\left[x_{0}|x_{t}\right]=x\left(t\right)+\sigma {\left(t\right)}^{2}\nabla_{x}\log\left(p\left(x\left(t\right)\right)\right)$. Since the second term resembles the trained score, the denoised estimate is computed in the case of VE SDEs by ${\hat{x}}_{0}=x\left(t\right)+\sigma {\left(t\right)}^{2}s_{\Theta }\left(x\left(t\right),\sigma \left(t\right)\right)$.

With $p\left(y|x_{0}\right)=\frac{1}{\sqrt{{\left(2\pi \right)}^{n}\sigma_{\tau }^{2n}}}\exp\left[-\frac{{\left\|y-Ax_{0}\right\|}_{2}^{2}}{2\sigma_{\tau }^{2}}\right]$ being the likelihood function for measuring *y*, given the clean $x_{0}$ with SD of the noise during measurement $\sigma_{\tau }$, the gradient of the log likelihood using the denoised estimate corresponds to $\nabla_{x_{t}}\log\left(p\left(y|{\hat{x}}_{0}\left(x_{t}\right)\right)\right)\approx -\frac{1}{\sigma_{\tau }^{2}}\nabla_{x\left(t\right)}{\left\|y-A{\hat{x}}_{0}\left(x\left(t\right)\right)\right\|}_{2}^{2}$. Plugging this into the conditioned score function results in $\nabla_{x}\log\left(p\left(x\left(t\right)|y\right)\right)\approx s_{\Theta }\left(x\left(t\right),t\right)-\gamma \;\nabla_{x\left(t\right)}{\left\|y-A{\hat{x}}_{0}\left(x\left(t\right)\right)\right\|}_{2}^{2}$, with γ being the step size. The gradient can now be determined using backpropagation for each timestep t as a part of solving the reverse SDE.

#### 2.3.3 Training for cardiac MR

To capture the probability distribution of cardiac MR cine images, the unconditioneddiffusion network $s_{\Theta }\left(x_{i},i\right)$ was trained using 2120 individual complex coil‐combined 2D spiral reference frames of size 2 × 512 px × 512 px, acquired in 96 slices of eight healthy participants (training data). Data were normalized with respect to the maximum value of the coil‐combined magnitude reconstruction of the 104 spiral arms, respectively (see Section 2.2).

The typical geometric series was used for the noise scheduler with $\sigma_{\min}=0.01$ and $\sigma_{\max}=378$. A total of N=2000 diffusion steps were trained using Adam optimizer with a batch size of 1 and a linear learning rate warm up, that reached a constant learning rate of $2\times 10^{-4}$ after 5000 steps with $\beta_{1}=0.9$ and $\beta_{2}=0.999$ and an exponential moving average rate of 0.999. The time‐dependent neural network called noise conditional score network ++ (ncsnpp) proposed by Song et al.^8^ was used as score function. This network receives a two‐channel input, corresponding to real and imaginary part of the complex coil‐combined image, as well as the time dependent noise scale, and delivers an output that estimates the complex noise superimposed on the input image.

**FIGURE 2:**
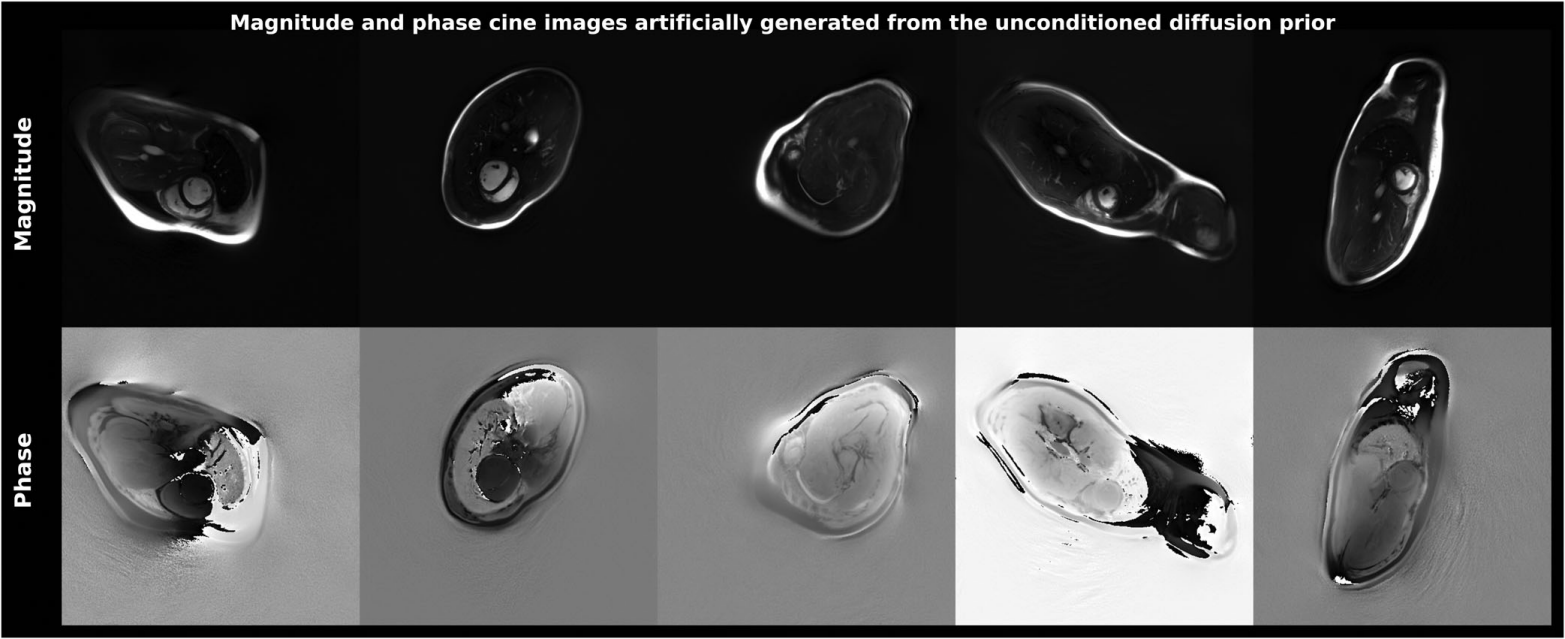
Artificial samples from the distribution of cardiac cine frames as provided by the complex trained diffusion model. Results overall comply with quality, contrast, field‐of‐view, gross anatomy, etc. of the original datasets and its variations. Deviations from realistic anatomy do not pose a fundamental problem for the downstream reconstruction scheme, as the data consistency term guides the diffusion process towards a result in accordance with the anatomy of the actual examination.

Training for 100 epochs took roughly 3 days on an RTX A6000 graphics processing unit (GPU) (48Gb). The diffusion model combined with the trained network is capable of generating realistically looking, artificial samples of the initial probability distribution (i.e. cine frames with contrast features etc. compliant with those of the training set, see Figure 2).

#### 2.3.4 Conditioned sampling for reconstruction of cardiac MR

To perform conditioned sampling from undersampled MR data, we employ the reverse diffusion sampler in combination with DPS as proposed by Chung et al.^31^ and discussed above. The iteration procedure is outlined by Algorithm 1. Similar to,^31^ we chose the step size schedule $\gamma_{i}=1/{\left\|y-A{\hat{x}}_{0}\left(x_{i}\right)\right\|}_{2}$ in the discrete implementation. As described in Section 2.1, data were initially transferred to Cartesian grids using GROG. Therefore, the forward operator A=MFS incorporates a coil decombination using the coil‐sensitivities S, a fast Fourier transform F and multiplication with the sampling mask M.

In the most straightforward execution, the algorithm would start by drawing a sample from pure Gaussian noise and running the reverse sampler for an extended number of timesteps. Alternatively, the procedure can be sped up by using a reconstruction estimate perturbed with noise from an earlier diffusion step for an initialization as outlined in.^20^, ^46^

We thus start with a complex coil‐combined temporal average image, normalized with respect to the maximum value of the respective magnitude image, additionally forward diffused with noise from step *n* = 100. By leveraging the information from a temporal average, no initial undersampling artifacts compromise the initialization; however, cardiac motion must be solely enforced through the DPS data consistency step.

We ran the diffusion reconstruction for the last *n* = 100 steps, with interleaved discretization steps by setting *N* = 500 during reconstruction, as suggested in.^20^ In the final step, no additional noise was added in the reverse diffusion sampler.

ALGORITHM 1 **DPS exploiting a diffusion probabilistic model**

Require: $s_{\Theta },n,{\left\{\sigma \right\}}_{i=1}^{n},\sigma_{0}=0,{\left\{\gamma \right\}}_{i=1}^{n},x_{\mathrm{avg}},y$

$x_{n}=x_{\mathrm{avg}}+\sigma_{n}N\left(0,I\right)$                    # Forward diffusion step

For *i* = *n*:1 do:

$x_{i-1}=x_{i}+\left(\sigma_{i}^{2}-\sigma_{i-1}^{2}\right)s_{\Theta }\left(x_{i},\sigma_{i}\right)$                # Reverse diffusion sampling

If *i* ! = 1: $x_{i-1}=x_{i-1}+\sqrt{\sigma_{i}^{2}-\sigma_{i-1}^{2}}\;N\left(0,I\right)$

${\hat{x}}_{0}\left(x_{i}\right)=x_{i}+\sigma_{i}^{2}s_{\Theta }\left(x_{i},\sigma_{i}\right)$                   # Estimate of the denoised image

$x_{i-1}=x_{i-1}-\gamma_{i}\nabla_{x_{i}}\;{\left|\left|y-A{\hat{x}}_{0}\left(x_{i}\right)\right|\right|}_{2}^{2}$               # DPS

End for

Return x0

**Caption:** $x_{\mathrm{avg}}$ corresponds to a temporally averaged coil‐combined image. $\sigma_{i}$ is the noise scale of the *i*‐th diffusion step, in our case starting from the step *n* = 100. Sampling with the trained score function $s_{\Theta }$ is performed using the reverse diffusion sampler. Diffusion Posterior Sampling is employed by computing an estimate of the denoised image at time *t* = 0 given by Tweedie's formula.

### 2.4 Variational networks

VNs represent an alternative and already more established data‐driven solution for the minimization problem in Eq. [1] and their performance has already been demonstrated for the reconstruction of undersampled MR,^9^ also for cardiac MRI with non‐Cartesian trajectories.^14^

For a comparative reconstruction of our undersampled spiral cMR data, we used an in‐house developed VN,^14^, ^47^ which is based on a cascaded U‐Net architecture. The unrolled blocks of the VN incorporated Kaiser‐Bessel–based gridding operations and coil‐(de)combinations using the torchkbnufft library,^48^ allowing to perform data consistency operations directly on the complex off‐grid raw data. For detailed implementation details we refer to^14^, ^47^. Therefore, the VN is the only model investigated in this study, which incorporates convolution gridding instead of GROG. Real and imaginary parts of the gridded coil‐combined images were normalized to zero‐mean and unit SD before feeding them as a two‐channel input into the network. A total of 20 cascades were used, with a total of four pooling layers and 16 channels in the first layer.

Training the VN for 13 epochs using the mean squared error (MSE)‐loss took ˜6 days on a single RTX A6000 GPU (48Gb). To this end, 16.960 undersampled timeframes from eight heartbeats from a total of 96 slices from the eight healthy volunteers of the training set were used together with the reference images from binned cine reference frames. A fixed learning rate of $5\times 10^{-3}$ was used for the Adam optimizer. As we want to use the VarNet as a best possible performing benchmark for assessing the diffusion model, we calculated scalar metrics for one healthy participant of the test set for all epochs and took the best performing epoch for all reconstructions then.

### 2.5 Model‐based reconstruction techniques – compressed sensing

Compressed sensing models assume sparsity of the data distribution in a specific representation space by integrating this prior knowledge as additional regularizer of the minimization problem. l1‐Total Variation (TV) and l1‐Wavelet (WV) were applied in this work to reconstruct real‐time frames of the test set separately (i.e., 2D reconstruction) for further comparison. The openly available BART‐toolbox^24^ was used for this purpose. Regularization parameters were set to λ=0.03 for both approaches (TV and WV). TV standardly employed ADMM optimization with 40 iterations, while WV reconstructions standardly used FISTA with 30 iterations. Prior to reconstruction, 2D multi‐coil raw data were normalized with respect to the maximum value of a naïve reconstruction (inverse Fourier transform of the data after applying GROG plus coil combination).

Additionally, we used an in‐house implementation of the low rank plus sparse (LRS) model that also enforces sparsity in the temporal domain.^23^ Here 60 iterations were performed using regularization values of $\lambda_{L}=\lambda_{s}=0.02$ for thresholding the singular values of the Casorati matrix (i.e. low‐rank approximation) and the temporal frequencies of the residual sparse domain, respectively. Data of the entire time series to be reconstructed were normalized with respect to the maximum value of the respective coil‐combined magnitude image series here.

### 2.6 Quantitative evaluation of image quality using scalar metrics

Ideally, a quantitative analysis of different reconstruction methods can be performed based on a fully sampled dataset that can be retrospectively undersampled. Because it is not feasible to acquire fully sampled “real‐time” frames, segmented cine can be used instead. Applying the breath‐held spiral acquisition scheme as depicted in Section 2.1, segmented spiral references can be reconstructed. To exclude temporal mismatches as much as possible, we retrospectively extracted an undersampled k‐space by applying the undersampling mask from a spiral real‐time acquisition to the spiral reference cine k‐space. Both were gridded using GROG. As the VN was based on convolution gridding rather than GROG, a (convolution) gridded reference was compared with respectively gridded real‐time undersampled reconstructions.

Retrospectively undersampled frames were generated using measurements from 94 slices of eight healthy volunteers. Data from two healthy volunteers were excluded (24 of 94 slices) for the final evaluation due to slight arrhythmia, ultimately leading to a failure of the automated data binning procedure and therefore clearly blurred references. Ultimately, a total of 1632 images were utilized. Segmented reference data as well as undersampled reconstructions were cropped to image sizes of 120 × 120 px, manually centered on the myocardium. Individual images in the time‐series were rescaled to a data range of [0 1].

We then evaluated the reconstruction methods described in 2.3–2.5 by computing the following metrics: structural similarity index (SSIM), normalized RMS error (NRMSE) and peak SNR (PSNR) and respective SDs using the skimage.metrics library.^49^

### 2.7 Expert reader study

Image quality of real‐time acquisitions in free‐breathing using a temporal series of 40 frames reconstructed by l1‐Wavelet, LRS, VN and diffusion, as well as breath‐held ECG‐gated Cartesian references were rated per subject by a board‐certified radiologist (J.F.H.; 7 years of cardiac imaging experience) in a blinded and fully randomized fashion. Data from 145 SAX oriented slices from eight healthy participants and five patients were analyzed. As data were pooled with (arrhythmic) patients anyway, we did not exclude the two volunteers with slight arrythmia. The rating was performed using ordinal five‐point rating scales for the following items: blood/myocardium contrast, temporal dynamics along the t‐dimension, sharpness of the myocardial contours (5: excellent, 4: good, 3: moderate, 2: fair, 1: poor), presence of noise which impairs assessment of myocardial structures and artifacts that affect the cardiac structures (5: none, 4: very few, 3: moderate, 2: substantial, 1: non‐diagnostic).

### 2.8 Quantification of functional parameters

To compare the reconstruction methods for cardiac function assessment, 145 SAX slices from five patients and eight healthy participants were manually segmented by an MD candidate under supervision, as well as the expert radiologist using a dedicated segmentation software for medical images (MEVIS draw, Frauenhofer MEVIS, Bremen, Germany). The real‐time free‐breathing acquisitions reconstructed by the diffusion approach were compared to the breath‐held Cartesian gold‐standard cine. In addition, segmented spiral cine reconstructions from breath‐held acquisitions were evaluated and compared with both the Cartesian cine and real‐time, free‐breathing diffusion reconstructions. Since slight blurring of segmented, spiral references presents no major impairment for this evaluation, the two volunteers previously excluded in Section 2.6, were also included here.

The following functional parameters were derived from manual segmentations: end‐systolic volume (ESV), end‐diastolic volume (EDV), stroke volume (SV) and ejection fraction (EF).

Differences in the quantification of functional parameters between ground truth (Cartesian cine), segmented spiral cine and the real‐time series were evaluated using Bland–Altman analysis.^50^, ^51^

### 2.9 Comparison of reconstruction time

In‐house implementations such as the VN, LRS, and the diffusion model were implemented and reconstructed on a GPU using the pytorch library,^52^ whereas the l1‐CS models ran on CPU only using BART.^24^ Reconstruction times were computed per frame. As LRS performs reconstruction for all frames together, total reconstruction times were divided by the number of reconstructed frames for comparison.

## 3 RESULTS

### 3.1 Qualitative comparison of diffusion model with alternative methods

**FIGURE 3:**
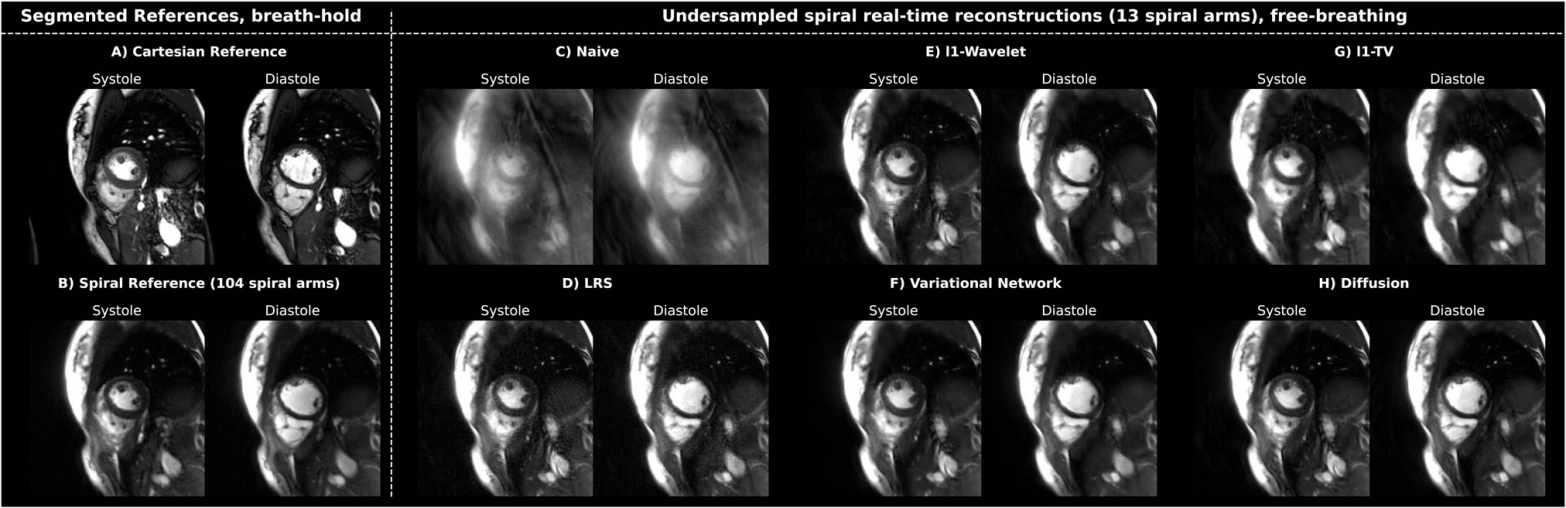
Overview of the different acquisition and reconstruction methods for a systolic and diastolic frame of one healthy participant in SAX orientation. (A) Images acquired by a Cartesian bSSFP protocol used in the clinical routine. Here, data are segmented over multiple heartbeats to reconstruct cardiac phases for a single (pseudo) heartbeat. (B) A similar averaging method can be applied to the spiral acquisition using “self‐gating” (see Section 2.1), resulting in the images shown. (C) Real‐time frames reconstructed by GROG only, and thus without additional regularization. (D–H) The images depict the results for different reconstruction methods applied to real‐time acquisitions in free‐breathing (temporal footprint 48 ms).

Figure 3 shows a comparison between different reconstruction approaches of a mid‐ventricular slice in a healthy participant in systolic and diastolic phase, respectively. The images on the left represent segmented/binned reconstruction (Cartesian and spiral) acquired in breath‐hold. All remaining images show reconstructions of undersampled real‐time frames in free‐breathing, with the respective technique indicated. Spiral acquisitions were cropped to match the FOV of the Cartesian reference.

As typical for bSSFP‐sequences, a strong contrast between the blood pool and the myocardium was obtained. Both segmented cine methods in Figure 3A,B) are free from apparent aliasing artifacts. For these acquisitions at 1.5T, bSSFP‐signal voids could efficiently be minimized by shimming and therefore are mainly observable in the outer regions of the anatomy. The gold‐standard Cartesian method provides a slightly higher contrast and resolution than the spiral method, which are caused by differences in the acquisition parameters, especially TE.

Increased sharpness of the Cartesian ECG‐gated reference might be due to off‐resonance effects, which cause mere image shifts in Cartesian sampling, but lead to spatial blurring in spiral trajectories.^53^ Especially the vessels in the lung appear clearer for the standard technique.

**FIGURE 4:**
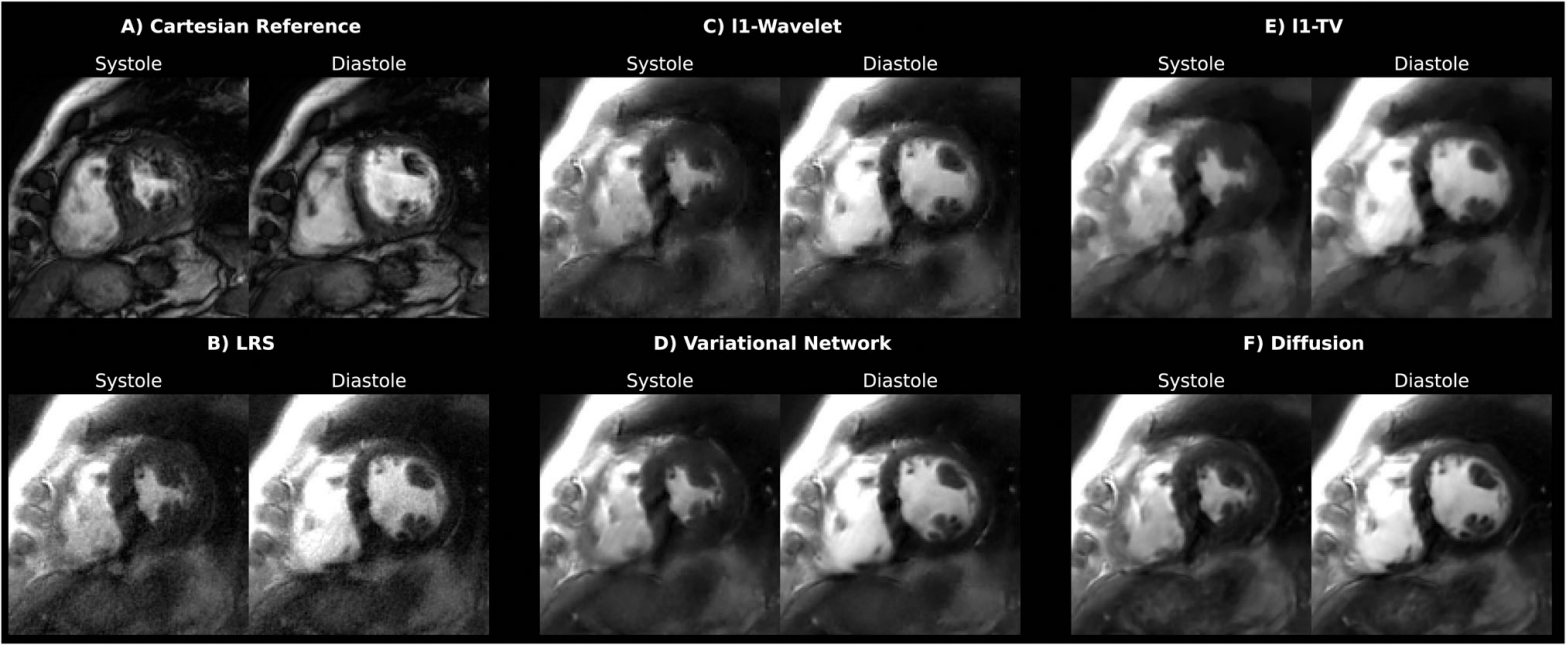
Comparison between the Cartesian reference (A) and different reconstruction techniques (B–F) applied to spiral acquisitions in real‐time free‐breathing for a systolic and diastolic frame of one arrhythmic patient in mid‐ventricular SAX orientation. Due to varying RR‐cycles, the Cartesian method was not capable of resolving cardiac phases accurately.

In patients with arrhythmia (see Figure 4), the segmented Cartesian method fails to correctly depict all cardiac phases due to temporal blurring induced by incorrect combination of data from different cardiac phases.

**FIGURE 5:**
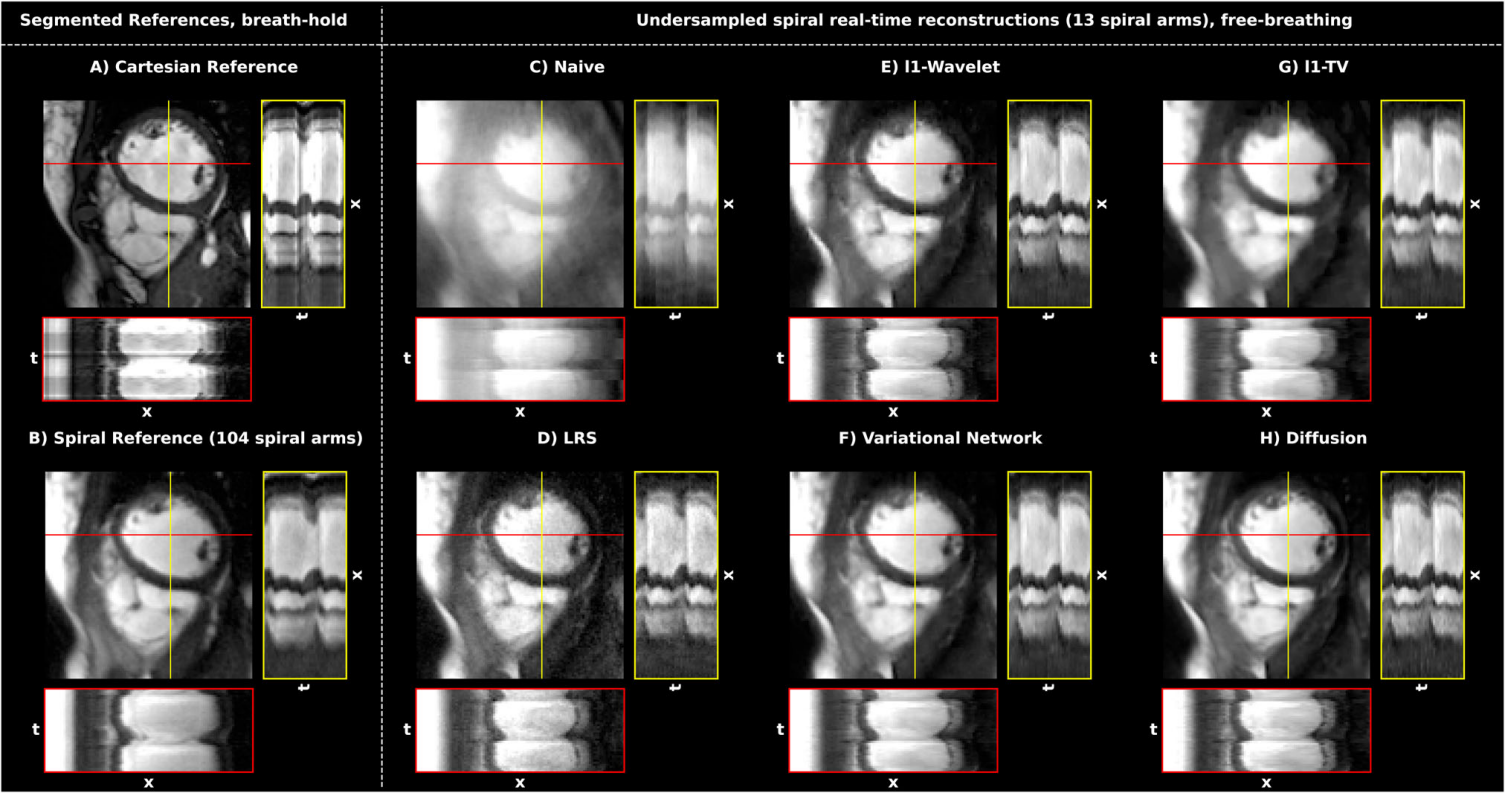
Spatio‐temporal examination of the diastolic frames as presented in Figure 3, centered on the myocardium. The x‐t plots represent 40 frames along the indicated vertical and horizontal lines. The Cartesian cine consisted of 21 frames, whereas the spiral cine contained 24 cardiac frames. The temporal evolution was partially repeated, respectively, to culminate in 40 frames. Visually, the diffusion reconstruction results in the sharpest depiction, especially also for the endocardial border.

For real‐time acquisitions in free‐breathing, all reconstruction methods are essentially able to largely remove undersampling artifacts (which are apparent in the naïve image reconstruction, Figure 3C). Differences between reconstructions methods can be seen more clearly in the cropped view depicted in Figure 5. While the l1‐TV method tends to spatially blur the image, the LRS method leads to an increase in noise. l1‐WV, VN, as well as the diffusion approach all offer high quality reconstructions, wherein both l1‐WV and VN seem to be slightly more blurred than the diffusion model. Also, fine structures appear to be the sharpest for the latter technique. Small signal fluctuations due to blood flow can be observed in the x‐t plots. At most, l1‐WV results in slight “Wavelet‐artifacts”, apparent as blocky clusters of pixels.

Videos S2 and S3 in Supporting Information show a comparison between reconstructions of a sequence of 40 timeframes of one healthy participant and one arrhythmic patient in mid‐ventricular SAX orientation. S4 and S5 in Supporting Information provide the corresponding results from diffusion‐based reconstruction of the real‐time acquisition in free‐breathing for the entire stack from base to apex. In the healthy volunteer, cardiac motion can be accurately displayed by the Cartesian reference as well as the real‐time reconstruction methods. In the patient, however, the segmented Cartesian acquisition fails and results in temporally blurred series, whereas the real‐time acquisition precisely depicts cardiac dynamics. Remaining artifacts from the pulsating blood flow are visible in all methods.

In the l1‐WV reconstruction, again, wavelet artifacts are apparent to a minor degree. Higher noise in LRS covers artifacts but also fine details. Slight blurring impacts the VN reconstructions. Contrast and spatial sharpness seem to be best for the diffusion model. In the temporal view, the stochastic nature of the reconstruction, which was performed for each frame individually led to marginal noise‐like artifacts in the time‐series. Furthermore, Video S6 illustrates nine cardiac phases from distinct subjects of the free‐breathing study, each repetitively reconstructed 50 times using the diffusion model. The present influence of stochastic variability arises as slight noise‐like flickering.

### 3.2 Quantitative comparison of reconstruction methods

**FIGURE 6:**
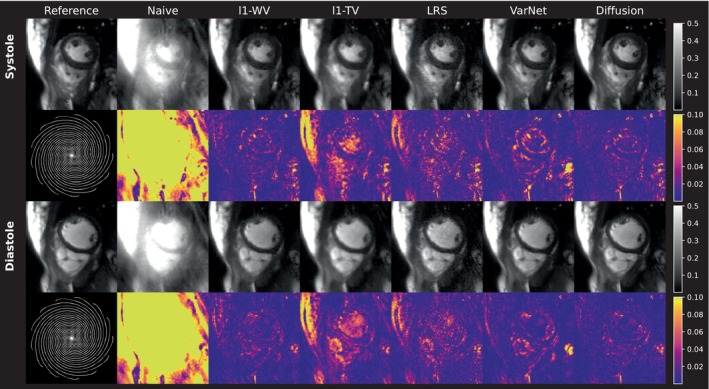
Simulation with retrospectively undersampled data. The undersampling pattern shown on the left side was applied to a systolic and diastolic frame, which holds segmented data acquired in breath‐hold across several heartbeats (i.e., Reference). The images obtained from applying the different reconstruction techniques to the resulting undersampled k‐spaces are depicted subsequently. Images were individually rescaled to a data range of [0 1]. The color encoded maps show absolute difference images between the reference and corresponding reconstructions.

Quantitative image quality metrics are summarized in Table 2. Difference images (Figure 6) highlight residual artifacts in the reconstructions of undersampled data: l1‐TV differences appear structural, while LRS differences resemble noise. Reconstructions using l1‐WV, VN, and diffusion methods appear qualitatively similar, though l1‐WV and VN show slightly more blurring compared to the diffusion method. This finding is supported by the quantitative metrics. Diffusion‐based reconstruction yielded the best overall SSIM, NRMSE, and PSNR‐values, although differences between methods fell within the respective SDs. Figure S1 shows slice‐wise metric distributions, including the 24 slices with blurred segmented, spiral references excluded from the mean values in Table 2. SSIM metrics performed considerably worse, with NRMSE also resulting in mean of values above 10%, deviating from the outcomes of the other subjects. Thus, Subjects 1 and 2 do not depict representative reconstruction results for the calculation of scalar metrics and where consequently excluded from the mean metrics depicted in Table 2.

**TABLE 2:** Results from the quantitative evaluation using scalar metrics.

Parameter	Naïve	l1‐TV	l1‐WV	LRS	VN	Diffusion
SSIM [%]	44.9 ± 4.5	90.5 ± 2.8	92.9 ± 2.1	88.9 ± 2.8	92.7 ± 2.7	93.5 ± 2.0
NRMSE [%]	107 ± 25	14.6 ± 6.8	8.6 ± 2.0	12.1 ± 3.5	9.0 ± 3.2	7.4 ± 1.6
PSNR [dB]	12.9 ± 1.6	30.8 ± 3.7	34.9 ± 2.4	32.0 ± 2.7	34.7 ± 3.2	36.1 ± 2.5

*Note*: SSIM, NRMSE, and PSNR with respective SD were calculated from 1632 cine frames from 70 slices of six healthy participants.

Table 3 presents the results from the randomized expert reader study. Differences among real‐time reconstruction methods were small, especially considering the SDs. Myocardium‐blood contrast and dynamics scores were largely independent of the reconstruction method. VN showed slightly reduced contrast, likely due to minor blurring. LRS reconstructions were noisier, while diffusion‐based reconstructions performed better in this regard. l1‐Wavelet reconstructions had more pronounced artifacts, whereas LRS received the best “Artifact” rating. This might be explainable either due to artifacts being suppressed by noise or a more efficient temporal information exploitation. Diffusion reconstructions yield slightly higher scores for “Sharpness of myocardial contours” compared to other methods.

**TABLE 3:** Results from the expert reader study.

Parameter	Cart. Cine	l1‐WV	LRS	VN	Diffusion
Myocardium‐blood contrast	5.0 ± 0	4.7 ± 0.5	4.7 ± 0.5	4.2 ± 0.5	4.8 ± 0.4
Noise	5.0 ± 0	4.1 ± 0.5	3.5 ± 0.5	4.0 ± 0.4	4.5 ± 0.5
Artifacts	4.6 ± 0.6	3.5 ± 0.8	4.4 ± 0.6	3.9 ± 0.3	3.9 ± 0.5
Sharpness of myocardial contours	3.6 ± 1.5	3.9 ± 0.6	4.0 ± 0.6	4.2 ± 0.4	4.6 ± 0.6
Dynamics	3.9 ± 1.1	4.9 ± 0.4	5.0 ± 0	4.8 ± 0.4	4.9 ± 0.3
Sharpness of myocardial contours ‐Patients only	2.4 ± 1.5	3.6 ± 0.5	4.0 ± 0.6	4.2 ± 0.4	4.6 ± 0.5
Dynamics ‐Patients only	3.0 ± 1.1	5.0 ± 0	5.0 ± 0	5.0 ± 0	4.8 ± 0.4

*Note*: Using the data of five patients and eight healthy participants, an expert reader study was performed on a 5‐point Likert‐scale (5: best, 1: worst) for the Cartesian cine as well as l1‐WV, LRS, VN, and diffusion real‐time reconstructions.

Notable differences emerged between the Cartesian gold‐standard and real‐time free‐breathing reconstructions. While the gold‐standard exhibits less noise and artifacts, dynamic motion can be significantly corrupted. In contrast, real‐time reconstructions received higher ratings for both “Dynamics” and “Sharpness” in depicting the cardiac movement especially when influenced by arrhythmia.

### 3.3 Cardiac functional parameters

**FIGURE 7:**
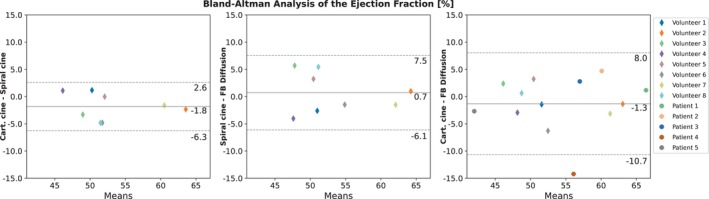
Bland–Altman analysis comparing the EF between the two segmented methods acquired in breath‐hold (“Cartesian cine” and “spiral cine”) and respective results for the diffusion reconstruction of real‐time, free‐breathing acquisitions. The horizontal lines depict mean differences and ± 1.96σ limits of agreement.

Figure 7 shows the results from the Bland–Altman analysis depicting the results from volumetry according to Section 2.8. EF shows overall good agreement between the three methods. There were no relevant differences in the absolute values of the EF between volunteers and patients. EF evaluation of the segmented Cartesian reference and binned spiral resulted in a small negative bias of −1.8 ± 4.5%. Analogously comparing segmented spiral cine from breath‐hold acquisitions with diffusion reconstruction of free‐breathing acquisitions resulted in 0.7 ± 6.8% mean differences of the EF. EF of Cartesian standard compared to free‐breathing real‐time depicts a bias of −1.3 ± 9.4%. However, separating healthy volunteers results in a bias of −1.1 ± 5.7%, while the isolated patient group depict a value of −1.6 ± 13%. Slight mean deviations are observable for EDV, ESV, and SV with 95% confidence intervals showing moderate uncertainties (as depicted in the full overview of the Bland–Altman analysis shown in Figure S2).

### 3.4 Speed of acquisition and reconstruction

The acquisition time of the Cartesian gold‐standard method in breath‐hold took between 4.0 and 10 s per slice. Spiral acquisitions were performed for 4.5–5.7 s in free‐breathing and up to 15 s in breath hold. Keep in mind, that long acquisition times in breath hold for the spiral acquisitions were only needed for the generation of reference data for training. Since real‐time acquisitions with a temporal resolution of 48 ms are possible with the proposed sequence and corresponding reconstruction techniques, the total acquisition time could be significantly reduced by acquiring data for roughly two heartbeats only. Without ECG‐gating and breath hold, the acquisition time, required for fully covering the whole heart would need less than 1 min with the spiral sequence.

Nevertheless, reconstruction times are still an issue for the advanced reconstruction used in this study. GROG of a temporal average k‐space took about 15.8 and 1.3 s for a single real‐time frame on CPU for ˜30 coils. Calculating coil sensitivity maps from the averaged k‐space took about 3.8 s. The fastest runtime of the subsequently applied algorithms was possible with the LRS model, which needed 0.4 s per frame with an implementation on a GPU. VN also offered fast reconstructions with 0.9 s for passing off‐grid data of one frame through the network. Since VN used GPU‐based convolution gridding only, no time‐consuming GROG was needed here. The diffusion approach took about 47 s for the reconstruction of one real‐time frame. Due to the large number of sampling steps with the additional need for gradient tracking, DPS also results in a heavier burden on GPU memory usage during inference in comparison to earlier methods that perform conditioning via a projection approach.^20^, ^21^ Both l1‐CS methods were restricted to run on CPU only, resulting in reconstruction times of 4.7 s for the l1‐TV and 3.8 s for the l1‐WV model. Moreover, the model‐based CS‐approaches do not need any time‐consuming training like the VN and the diffusion model.

## 4 DISCUSSION

In this work, we aimed to investigate the potential of exploiting a diffusion probabilistic model for the reconstruction of undersampled “real‐time” cardiac cMRI with spiral readouts. To this end, a score‐based diffusion model was first trained with cardiac cine images and then integrated into a physics‐based reconstruction scheme. The performance of this approach was compared to various state‐of‐the‐art model‐based and data‐driven reconstruction techniques such as l1‐compressed sensing (total variation and wavelet),^24^ low rank plus sparse,^23^ and a VN.^14^ While the Cartesian gold‐standard offered the highest image quality in the case of regular heartbeats, binning data is susceptible to allocation errors in arrhythmic or non‐compliant patients. In this regard, we showed that the proposed imaging technique facilitates an alternative approach to the clinical standard, offering comparable temporal and spatial resolution and, ultimately, allowing robust evaluation of cardiac functional parameters. In general, real‐time cMRI in free‐breathing can significantly increase patient comfort, reduce costs and indirectly increase accessibility of this high‐quality examination to a broader collective.

### 4.1 Stochastic variability

Even though data consistency is iteratively enforced for diffusion posterior sampling, the stochastic nature of diffusion models can lead to varying results for repeated reconstructions, which presents a concern for clinical translation. In our study, we did not detect any impairing hallucinations, but only a slight noise in the dynamic view of the repeated reconstructions (see Video S6). Uncertainty maps, as used in previous studies^21^ represent an alternative means to provide a clear depiction of these effects.

### 4.2 Reconstruction speed

One major disadvantage of current implementations of probabilistic diffusion models are certainly the demanding computational efforts and long inference times, as the reverse diffusion process is typically based on several hundreds of time steps. Inspired by Chung et al.,^46^ we did not initialize the reconstruction with pure Gaussian noise, but with a preliminary reconstruction provided by a temporally averaged estimate, to justify a significant reduction of the number of “iterations”. Different from the approach of Chung and Ye,^20^ we perform sampling on coil‐combined images, to avoid time consuming diffusion sampling for each coil separately. During conditioning, GROG was used to move data to a Cartesian grid once, thereby avoiding a gridding‐bottleneck throughout the reverse time steps. Nevertheless, latencies of ˜47 s for a single frame are too long to provide meaningful delays for clinical routine^54^ and more conceptual approaches to speed up the reconstruction are needed.

Denoising Diffusion Implict Models (DDIM) were proposed as a non‐Markovian, deterministic alternative approach to traditional diffusion models. Adaptions of the DDIM framework for the reconstruction of undersampled MR data were adapted in,^55^, ^56^ achieving high image‐qualities with reduced sampling steps and accelerated sampling times.

Distillation techniques might also be applicable in the context of diffusion models. Here the key idea is to train two separate models, namely a teacher model, which presents the complex iterative diffusion sampling process and student model, which aims to replicate the teacher model in a more efficient way, consequently trading reconstruction speed for reconstruction quality. Especially initialization plays a crucial role to accelerate diffusion inference times. Fourier diffusion bridges (FDB) start the diffusion chain with an initial state and transition faster to the target distribution by including structured noise into a generalized diffusion model. Early application of FDBs for accelerated MR reconstruction have also been shown in.^57^ Korkmaz et al.^58^ proposed an unrolled approach to realize self‐supervised MRI reconstruction using only five inference steps with a transformer architecture. The significant acceleration was here also made possible by initializing the reconstruction with a zero‐filled estimate. In Güngör et al.,^36^ the authors employed a large diffusion step size for rapid sampling. Since traditional diffusion models assume normality for the reverse transition, the authors employ an additional network to implicitly represent the distribution of a reverse diffusion step. Similarly, Zhao et al.^59^ combine diffusion sampling and GANs to achieve state‐of‐the‐art reconstruction performance using only 16 diffusion steps.

While our work focused on developing “real‐time” acquisitions that depict good image quality at a high temporal resolution for a dedicated point in time, authors of similar work partially restrict the term “real‐time” to techniques that furthermore reduce addressed reconstruction latencies to a few hundred milliseconds after acquisition.^54^ We also depicted reconstruction times for GPU‐accelerated and solely CPU‐based methods, which is definitely limited. We mainly intended to present an overview of reconstruction times during this study and also recognize that further optimizations may significantly reduce latencies.

### 4.3 Evaluation of image quality and Bland–Altman analysis

Compared to alternative methods one advantage of the diffusion model was the ability to reconstruct somewhat sharper images with higher SNR, as indicated by the results of the quantitative metrics and expert reader study. In particular, when compared to the images obtained from corrupted ECG‐gated acquisitions in case of arrhythmia, VN‐ and diffusion‐based reconstructions of real‐time data appear to be of comparable high quality.

However, the classical scalar metrics SSIM, NRMSE, and PSNR are not really predestined to work out subtle image quality differences sensitively. With no fully sampled ground‐truth data available for a single real‐time frame, our calculations were based on a segmented spiral reference with data from multiple heartbeats. Slight variations in the heart rate may result in blurring and a loss of detail in the reference data. Consequently, this leads to reduced metrics for methods that preserve details while improving metrics for ones that exhibit slight blurring. Moreover, it has also been indicated that SSIM, PSNR, and NRMSE do not necessarily fully capture the visual perceived quality and other metrics might be more suitable for this task.^60^ With these quantitative metrics often solely being used for the evaluation of image quality, this discrepancy presents an important issue.

Volumetric evaluation using Bland–Altman analysis showed on average agreement of Cartesian gold‐standard and free‐breathing real‐time acquisition reconstructed using the diffusion model. Nevertheless, significant uncertainties occurred, limiting conclusion that can be inferred from the values. For the segmented methods, such as the Cartesian standard and the segmented spiral acquisitions, only a singular heartbeat is available. Here, averaging effects might influence the evaluation of the endocardial contour in end‐systolic cardiac phase, leading towards lower results with respect to the EF. Variability in the dynamic motion in different cardiac cycles could also affect the evaluation of the real‐time approach. Even though multiple heartbeats were acquired, only the endocardial border of a single end‐diastolic/end‐systolic border was segmented by the examiner due to the extensive effort involved. However, segmenting multiple real‐time heartbeats might also allow to evaluate the variations in the cardiac motion. Additionally, physical load due to breath‐hold acquisitions versus free‐breathing measurements might also influence volumetric parameters. A proper comparison would therefore necessitate a clinically validated real‐time method, which was not accessible during the study period. Lastly, segmentations were performed by two examiners, introducing inter‐variability as well as intra‐variability in the evaluation.

We furthermore acknowledge that the evaluation dataset containing eight healthy volunteers and five patients is rather small and a more rigorous analysis would profit from a larger subject cohort.

### 4.4 Hyperparameters of diffusion models

An accelerated inference was achieved by initializing the diffusion model with a coarse temporally averaged reconstruction and consequently a reduction of the number of diffusion steps, similarly to Chung et al.^46^ In general, noise scheduler, number of perturbation steps, data consistency weighting, initialization, number of diffusion steps during inference, and, not least, the loss function all influence the final reconstruction quality. Rigorous optimization of these factors as well as the exploration of various network architectures might further yield improved overall performance, but was beyond the scope of the current study.

Diffusion‐based reconstruction methods are capable of improving image quality, further leveraging the capabilities of accelerated MRI. Nevertheless, differences between established data‐driven methods, such as the VN were low, which does not currently justify the great effort involved in training and the comparatively long computing times.

Due to the very promising and versatile properties also shown in this paper, diffusion models in medical imaging are definitely on the rise^16^, ^32^ and intensive investigations, regarding improved inference speed and image quality might resolve their current obstacles.

## 5 CONCLUSIONS

We proposed a diffusion probabilistic model to reconstruct undersampled real‐time cardiac cine MRI based on spiral trajectories acquired in free‐breathing as a promising strategy to provide shorter and more robust cMRI exams, especially in patients with irregular cardiac cycles and who cannot hold breath properly. In a comparison with alternative state‐of‐the‐art reconstruction techniques, the diffusion model showed potential to improve image quality, however, at the cost of prolonged inference times and slight stochastic variability with the current implementation.

## FUNDING INFORMATION

This work was supported by the Bundesministerium für Bildung und Forschung under Research Grant 05M20WKA and the Interdisciplinary Center for Clinical Research in Würzburg under Research Grant F‐437.

## Supporting information


**Figure S1.** Quantitative metrics computed from the retrospectively undersampled segmented spiral cine acquisitions, reconstructed with the indicated reconstruction methods. Dashed vertical lines separate data from the eight healthy subjects. Due to temporal blurring of spiral references, data from the first two subjects (first 24 slices) were discarded in the computation of the metrics in the main text.

**Figure S2.** Full overview of the Bland–Altman Analysis showing ejection fraction, end‐diastolic volume, end‐systolic volume and stroke volume. Comparison of volumetric volumes were performed for the clinical Cartesian cine vs. the segmented spiral cine acquired in breath hold, as well as for the prospective free‐breathing real‐time diffusion reconstructions versus spiral cine and Cartesian cine. Separate evaluations of the healthy subject group and the patient group are shown in the last two rows.

**Video S1.** Schematic animation depicting the acquisition procedure of breath‐held, segmented spiral cine.

**Video S2.** Left: ECG‐gated Cartesian cine and a “aliasing free” segmented spiral acquisition acquired in breath‐hold. Right: Reconstructions of undersampled real‐time frames acquired in free‐breathing and reconstructed using wavelet‐ and total variation‐based compressed sensing, low rank plus sparse, a variational network and a score‐based diffusion model. The time series shows 40 frames acquired in a healthy volunteer in midventricular short‐axis orientation.

**Video S3.** Top‐left: ECG‐gated Cartesian cine acquired in breath‐hold. Bottom‐left & right: Reconstructions of undersampled real‐time frames acquired in free‐breathing and reconstructed using wavelet‐ and total variation‐based compressed sensing, low rank plus sparse, a variational network and a score‐based diffusion model. The time series shows 40 frames acquired in a patient with irregular RR‐cycles in midventricular short‐axis orientation.

**Video S4.** 40 real‐time frames showing 12 slices of a healthy volunteer covering the whole heart using the real‐time spiral acquisition scheme in free‐breathing. Undersampled acquisitions were reconstructed using a score‐based diffusion model.

**Video S5.** 40 real‐time frames showing 11 slices of a patient with irregular RR‐cycles covering the whole heart using the real‐time spiral acquisition scheme in free‐breathing. Undersampled acquisitions were reconstructed using a score‐based diffusion model.

**Video S6.** Overview of nine cardiac phases from distinct subjects of the free‐breathing study, each repetitively reconstructed 50 times using the diffusion model. The present influence of stochastic variability in the reconstruction arises as slight noise‐like flickering.

## Data Availability

The source code of the presented diffusion model is publicly available at https://github.com/expRad/Diffusion.
