# Supplementary material for: Real‐time cardiac cine MRI: A comparison of a diffusion probabilistic model with alternative state‐of‐the‐art image reconstruction techniques for undersampled spiral acquisitions
Source: Magn Reson Med. 2025 Jun 16;94(4):1731–49. doi: 10.1002/mrm.30572 (PMC12309890; doi:10.1002/mrm.30572)
Supplement: Supplementary file 1 — Figure S1. Quantitative metrics computed from the retrospectively undersampled segmented spiral cine acquisitions, reconstructed with the indicated reconstruction methods. Dashed vertical lines separate data from the eight healthy subjects. Due to temporal blurring of spiral references, data from the first two subjects (first 24 slices) were discarded in the computation of the metrics in the main text. [file MRM-94-1731-s007.pdf]

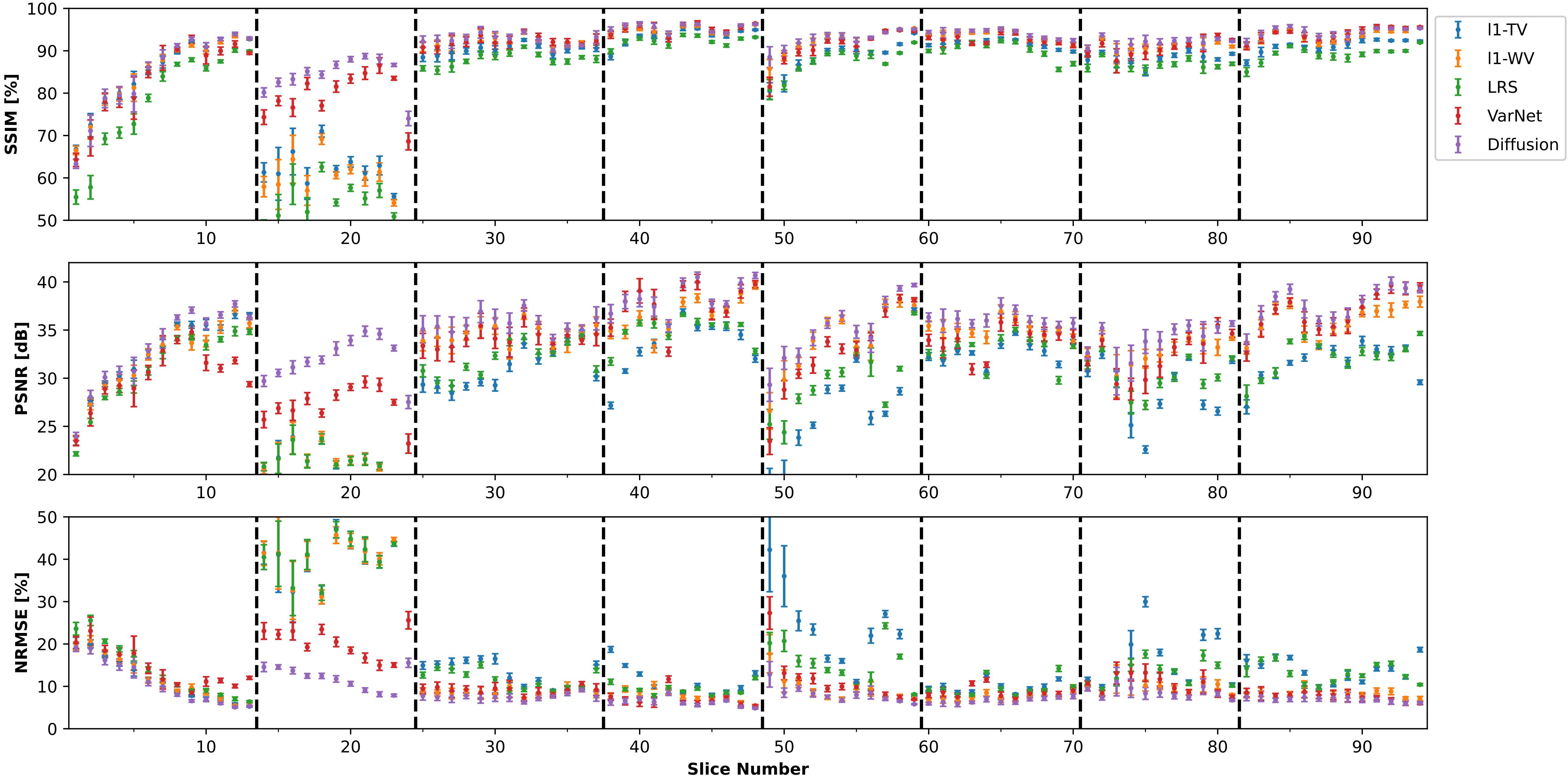


Figure S1: Quantitative metrics computed from the retrospectively undersampled segmented spiral cine acquisitions, reconstructed with the indicated reconstruction methods. Dashed vertical lines separate data from the 8 healthy subjects. Due to temporal blurring of spiral references, data from the first two subjects (first 24 slices) were discarded in the computation of the metrics in the main text.
