# Supplementary material for: Real‐time cardiac cine MRI: A comparison of a diffusion probabilistic model with alternative state‐of‐the‐art image reconstruction techniques for undersampled spiral acquisitions
Source: Magn Reson Med. 2025 Jun 16;94(4):1731–49. doi: 10.1002/mrm.30572 (PMC12309890; doi:10.1002/mrm.30572)
Supplement: Supplementary file 2 — Figure S2. Full overview of the Bland–Altman Analysis showing ejection fraction, end‐diastolic volume, end‐systolic volume and stroke volume. Comparison of volumetric volumes were performed for the clinical Cartesian cine vs. the segmented spiral cine acquired in breath hold, as well as for the prospective free‐breathing real‐time diffusion reconstructions versus spiral cine and Cartesian cine. Separate evaluations of the healthy subject group and the patient group are shown in the last two rows. [file MRM-94-1731-s005.pdf]

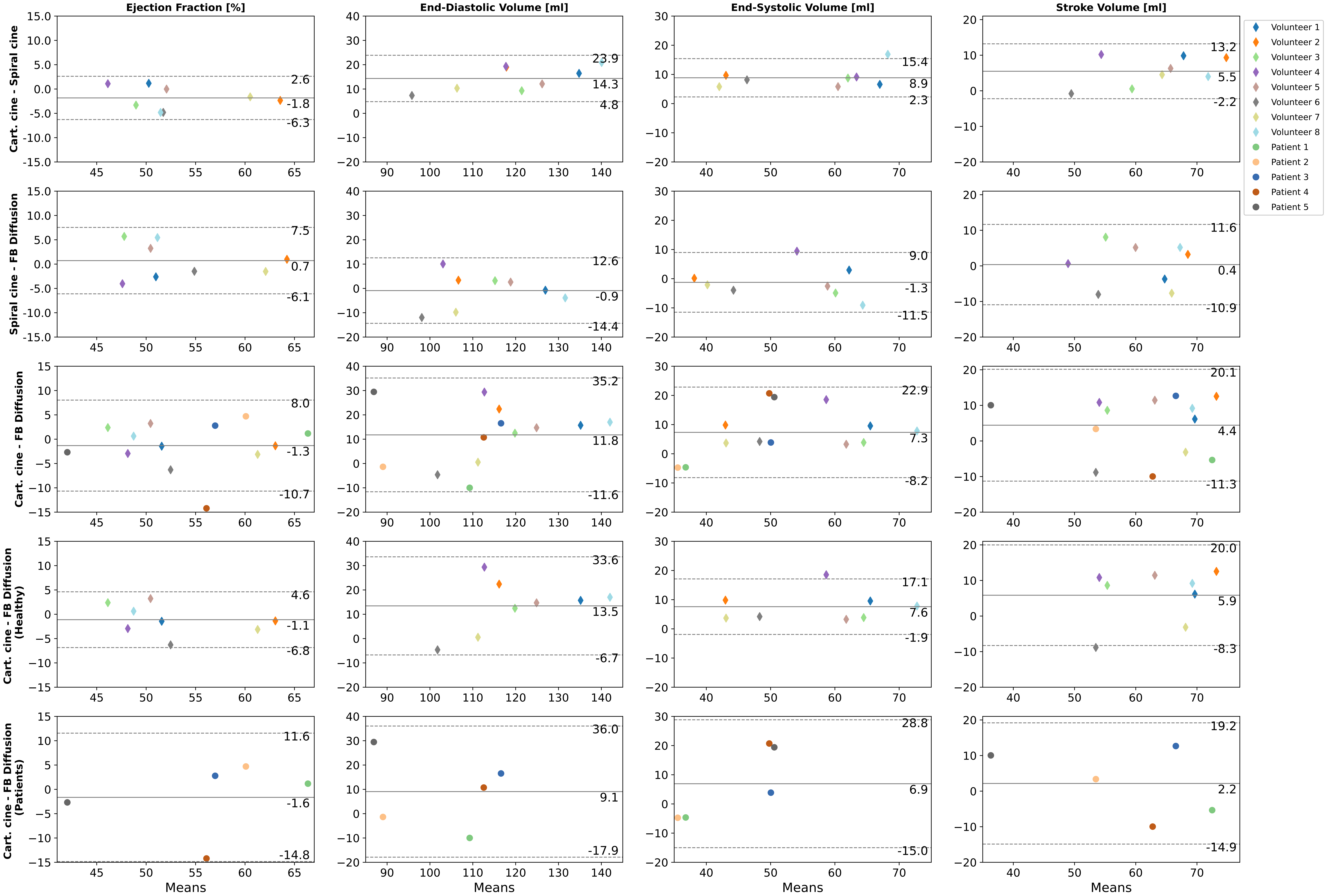

Figure S2: Full overview of the Bland-Altman Analysis showing ejection fraction, end-diastolic volume, end-systolic volume and stroke volume. Comparison of volumetric volumes were performed for the clinical Cartesian cine vs. the segmented spiral cine acquired in breath hold, as well as for the prospective free-breathing real-time diffusion reconstructions vs. spiral cine and Cartesian cine. Separate evaluations of the healthy subject group and the patient group are shown in the last two rows.
